# Hydrothermal and Entropy Investigation of Nanofluid Mixed Convection in Triangular Cavity with Wavy Boundary Heated from below and Rotating Cylinders

**DOI:** 10.3390/nano12091469

**Published:** 2022-04-26

**Authors:** Bellakhdar Mohamed Cherif, Aissa Abderrahmane, Abdulkafi Mohammed Saeed, Naef A. A. Qasem, Obai Younis, Riadh Marzouki, Jae Dong Chung, Nehad Ali Shah

**Affiliations:** 1 Laboratoire de Physique Quantique de la Matière et Modélisation Mathématique (LPQ3M), University Mustapha Stambouli of Mascara, Mascara 29000, Algeria; cherifdaoud57@gmail.com (B.M.C.); a.aissa@univ-mascara.dz (A.A.); 2 Department of Mathematics, College of Science, Qassim University, P.O. Box 6644, Buraydah 51452, Saudi Arabia; abdulkafi.ahmed@qu.edu.sa; 3 Department of Aerospace Engineering & Interdisciplinary Research Center for Membranes and Water Security, King Fahd University of Petroleum & Minerals (KFUPM), Dhahran 31261, Saudi Arabia; naefqasem@kfupm.edu.sa; 4 Department of Mechanical Engineering, College of Engineeing at Wadi Addwaser, Prince Sattam Bin Abdulaziz University, Wadi Addwaser 11991, Saudi Arabia; oubeytaha@hotmail.com; 5 Chemistry Department, College of Science, King Khalid University, Abha 61413, Saudi Arabia; rmarzouki@kku.edu.sa; 6 Department of Mechanical Engineering, Sejong University, Seoul 05006, Korea; jdchung@sejong.ac.kr

**Keywords:** three-dimensional triangular cavity, magnetic field, hybrid nanofluids, porous media, heat transfer, undulation wall

## Abstract

Nanofluids have become important working fluids for many engineering applications as they have better thermal properties than traditional liquids. Thus, this paper addresses heat transfer rates and entropy generation for a Fe_3_O_4_/MWCNT-water hybrid nanoliquid inside a three-dimensional triangular porous cavity with a rotating cylinder. The studied cavity is heated by a hot wavy wall at the bottom and subjected to a magnetic field. This problem is solved numerically using the Galerkin finite element method (GFEM). The influential parameters considered are the rotating cylinder speed, Hartmann number (Ha), Darcy number (Da), and undulation number of the wavy wall. The results showed that higher Da and lower Ha values improved the heat transfer rates in the cavity, which was demonstrated by a higher Nusselt number and flow fluidity. The entropy generation due to heat losses was also minimized for the enhanced heat transfer rates. The decrease in Ha from 100 and 0 improved the heat transfer by about 8%, whereas a high rotational speed and high Da values yield optimal results. For example, for Ω = 1000 rad/s and Da = 10^−2^, the enhancement in the average Nusselt number is about 38% and the drop in the Bejan number is 65% compared to the case of Ω = 0 rad/s and Da = 10^−5^. Based on the applied conditions, it is recommended to have a high Da, low Ha, one undulation for the wavy wall, and high rotational speed for the cylinder in the flow direction.

## 1. Introduction

According to recent research, suspending solid nanoparticles in conventional working fluids might increase heat transfer rates by enhancing thermal conductivity and heat transfer coefficients. However, the extent to which heat transfer increases are described in the literature varies considerably [1,2,3,4]. Numerous research papers (both experimentally and numerically) have been published on the topic of heat transmission and the flow of nanofluids [5,6,7]. Sajid et al. [8] offered an in-depth study of the use of nanofluids in a variety of heat transfer applications, including heat exchangers, heat sinks, and radiators. Kannan et al. [9] compared the cooling properties of pure water and Al_2_O_3_ nanoliquids. The concentration of nanoparticles was the most influential parameter affecting heat transmission rate compared to flow rate and inlet temperature since thermal conductivity is directly proportional to nanoparticle concentration, which is the primary factor for this increase in heat transmission rate. Nazari et al. [10] cooled the CPU using a variety of coolants. CNT nanofluid was shown to be superior to ethylene glycol, water, and alumina nanoliquid as a coolant. Vijayakumar et al. [11] demonstrated that the optimal nanofluid volume fraction in a heat pipe is reliant on the nanoparticle type and that the thermal resistance of the heat pipe decreases as the concentration of nanofluids increases. Saeed et al. [12] raised the amount of distilled water produced to 43% by using a CuO2/water nanofluid at a mass concentration of 0.08 percent in the condensing channel of thermoelectric cooler modules in a solar still. Adnan et al. [13] stated that the concentration of nanoparticles and the friction factor are proportional and that the inlet temperature has no discernible influence on the friction factor. For 2.5 vol% concentrated nanofluid, the largest growth in friction factor was 22%. Another study [14] demonstrated a maximum increase in energy rate and efficacy of around 32 and 29.5%, respectively, for SiO_2_/water and TiO_2_/water nanofluids. They also detailed that the SiO_2_/water nanofluid performed better than TiO_2_/water nanofluid owing to the lower size of SiO_2_ nanoparticles.

Recently, more attention has been paid to the impact of both spinning cylinders and porous media on the mixed convection motion of nanoliquid, as both of these approaches are excellent for controlling the motion and movement of energy in a wide variety of thermal systems. Barnoon et al. [15] examined the influence of magnetism on mixed convection and entropy production in an Al_2_O_3_/water-loaded chamber with two spinning cylinders. According to their results, the increments in both the nanoparticles volume fraction and the angular rotating velocity boosted the Nusselt number. The reverse effect is observed when the Richardson and Hartmann coefficients are increased. Siavashi et al. [16] conducted an examination of mixed convection in a partially permeable container occupied with non-Newtonian CuO/H_2_O-based nanofluid and containing a spinning inner cylinder. They determined heat transmission may be enhanced by boosting convection or decreased by raising viscosity. Selimefendigil et al. [17] employed the finite element technique to model convection in a 3D reservoir full of a variety of nanofluids and including two adiabatic spinning cylinders. Alsabery et al. [18] investigated an Al_2_O_3_/water nanofluid-loaded hollow with a wavy wall and containing a revolving conductive cylinder. The findings indicated that the rotational velocity had a detrimental influence on thermal performance when the Rayleigh number was larger than or equal to 5*10^5^. Selimefendigil et al. [19] simulated the mixed convection caused by an inner revolving adiabatic tube in a 3D enclosure with differentially heated and wavy walls and loaded with SWCNT/water nanofluid. The surface corrugation parameter has a negligible impact on thermal performance with respect to the rotation speed and solid volume fraction. Jasim et al. [20] explored the concept of an internal adiabatic spinning cylinder within a vented chamber loaded with a hybrid nanofluid. The findings demonstrate that the transfer of energy of hybrid nanofluids rises with an increment in the solid particles’ volume percentage, but regrettably, a greater pressure drop is also seen. Chatterjee et al. [21] explored mixed convective motion inside an enclosure loaded with copper/water nanoliquid and including a revolving adiabatic cylinder. The upper wall was supposed to be cold and move to the right side, while the lower wall was kept immobile and heated. It was discovered that the Nusselt number grew as the nanoliquid concentration improved and the cylinder’s rotating speed declined. Selimefendigil et al. [22] explored the MHD convection of a CuO/water nanoliquid in an enclosure, taking into account the impacts of surface waviness and an interior spinning hot cylinder. It was determined that expanding the number of corrugations decreased the Num, while increasing the angular rotational speed. Darcy’s law is often utilized to describe the flow occupying the porous gap. Darcy’s notion remains inaccurate for collisions with high velocity and turbulence in porous media. To account for the effect of inertia on relative permeability, Forchheimer [23] included a second-order polynomial into the momentum formula. Muskat [24] designated it as the Forchheimer component. Recently, the non-Darcian effect was included in the examination of convective transport in a porous medium. Due to its importance in a variety of sectors, it is necessary to have a thorough grasp of convective heat transmission from a heated surface immersed in a porous medium occupied with a nanofluid as the working fluid [25,26,27,28]. Srinivasacharya et al. [29] examined the impact of radiation on a mixed convective flow in a non-Darcy porous medium loaded with nanoliquid and having an inclined wavy surface. Radiation was shown to improve the rate of local heat and mass transmission in both supporting and counterflow situations. Hayat et al. [30,31] inspected the influence of MHD and Darcy–Forchheimer on the entropy production of viscous fluid flow across a stretched sheet using thermal flux and joule heating. Kumar et al. [32] performed a numerical analysis of the heat transmission and flow of CNTs nanofluid flowing in divergent and convergent channels and exposed to heat radiation. Khan et al. [33] investigated the impact of activation energy on viscous fluid stagnation point flow using Darcy–Forchheimer relations and multi slips. Rasool et al. [34] established effective mathematical modeling to be used for designating the creation of entropy and the influences of binary chemical reactions on MHD Williamson nanoliquid flow in a porous medium across a nonlinearly expanding flat sheet in a Darcy–Forchheimer scenario. The findings indicated that a higher Weissenberg number increased the Bejan number. Chakraborty et al. [35] inspected the influence of solar energy on an Ag–water nanofluid flow through a sloping porous plate placed within a non-Darcy porous media. The increasing solar radiation factor resulted in a rise in the height of the momentum boundary layer. Kumar et al. [36] examined the relative model for MHD three-dimensional flow of Casson nanoliquid and Carreau nanoliquid currents caused by a moving flat form in a Darcy–Forchheimer medium, taking Soret and Dufour influences into consideration. Saif et al. [37] reported on the streams of nanofluids across a Darcy–Forchheimer porous medium. The simulation outcomes display that variations in the fluid flow resulted in the formation of a stretchy curve surface.

As a result of the previous study, this article aims to explore the mixed convection inside a 3D triangular cavity equipped with an internal rotating cylinder and bottom wavy wall and loaded with a porous medium saturated with hybrid nanofluids. A hybrid nanofluid is established when two distinct nanoparticles, Fe_3_O_4_ and MWCNT, are suspended in pure water. The impact of various factors, i.e., Darcy number, Hartmann number, cylinder speed, and number of wavy-wall undulations, on the flow and heat transfer characteristics is represented in 3D visualizations and 2D profile plots in the light of streamlines, temperature isotherms, Nusselt number, and entropy generation contribution by heat transfer. The findings of this study will assist researchers in acquiring a better grasp of the effects of spinning cylinders inside cavities and expand the usage of sophisticated triangular geometries in their future research.

## 2. Mathematical Model

**Figure 1:**
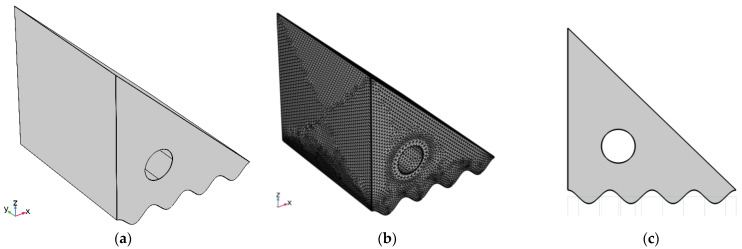
The computational domain (**a**) 3D view of the chamber, (**b**) 2D view of the geometry and (**c**) grid mesh.

The presented convective motion in this study is considered to be steady, 3D, and laminar. Figure 1 displays the computational domain, which comprises porous media saturated with hybrid nanoliquid, as well as the boundary conditions associated with it. The vertical wall and cylinder are adiabatic, but the bottom wavy wall and inclined wall are heated and cooled, respectively. A variety of patterns for the bottom wavy wall are studied in order to determine the influence of enclosure geometry. It is also taken into consideration that magnetic field effects exist by applying a constant magnetic field along the positive *Z*-axis. Water is used as the base liquid for the working suspensions and mixed with Fe_3_O_4_ and MWCNT nanoparticles to generate the suspensions. The thermophysical characteristics of both the base fluid and the nanoparticles are summarized in Table 1.

The conservation equations for mass, momentum, and energy, and also the entropy generation equation, may be written as follows in a two-dimensional Cartesian coordinate system [40,41,42,43]:

The conservation equations (CEs) are expressed in the porous area.

(1) $$\frac{\partial U}{\partial X}+\frac{\partial V}{\partial Y\;}+\frac{\partial W}{\partial Z}=0$$

(2) $$\frac{\rho_{\mathrm{nf}}}{\rho_{f}}\left[\frac{U}{\varepsilon^{2}}\frac{\partial U}{\partial X}+\frac{V}{\varepsilon^{2}}\frac{\partial U}{\partial Y}+\frac{W}{\varepsilon^{2}}\frac{\partial U}{\partial Z}\right]=-\frac{\rho_{\mathrm{nf}}}{\rho_{f}}\frac{\partial P}{\partial X}+\frac{1}{Re}\frac{1}{\varepsilon }\frac{\mu_{nf}}{\mu_{f}}\left(\frac{\partial U}{\partial X}+\frac{\partial U}{\partial Y}+\frac{\partial U}{\partial Z}\right)-\frac{\mu_{nf}\;\;\;}{\mu_{f}ReDa}U-\frac{\rho_{nf}}{\rho_{f}}\frac{C_{F}}{\sqrt{Da}}\sqrt{U^{2}+V^{2}+W^{2}}\;U$$

(3) $$\frac{\rho_{\mathrm{nf}}}{\rho_{f}}\left[\frac{U}{\varepsilon^{2}}\frac{\partial V}{\partial X}+\frac{V}{\varepsilon^{2}}\frac{\partial V}{\partial Y}+\frac{W}{\varepsilon^{2}}\frac{\partial V}{\partial Z}\right]=-\frac{\rho_{\mathrm{nf}}}{\rho_{f}}\frac{\partial P}{\partial Y}+\frac{1}{Re}\frac{1}{\varepsilon }\frac{\mu_{nf}}{\mu_{f}}\left(\frac{\partial V}{\partial X}+\frac{\partial V}{\partial Y}+\frac{\partial V}{\partial Z}\right)-\frac{\mu_{nf}}{\mu_{f}ReDa}V-\frac{\rho_{nf}}{\rho_{f}}\frac{C_{F}}{\sqrt{Da}}\sqrt{U^{2}+V^{2}+W^{2}}V-\frac{\sigma_{nf}}{\sigma_{f}}Ha^{2}\;\frac{V}{\varepsilon }$$

(4) $$\frac{\rho_{\mathrm{nf}}}{\rho_{f}}\left[\frac{U}{\varepsilon^{2}}\frac{\partial W}{\partial X}+\frac{V}{\varepsilon^{2}}\frac{\partial W}{\partial Y}+\frac{W}{\varepsilon^{2}}\frac{\partial W}{\partial Z}\right]=-\frac{\rho_{\mathrm{nf}}}{\rho_{f}}\frac{\partial P}{\partial Z}+\frac{1}{Re}\frac{1}{\varepsilon }\frac{\mu_{nf}}{\mu_{f}}\left(\frac{\partial W}{\partial X}+\frac{\partial W}{\partial Y}+\frac{\partial W}{\partial Z}\right)-\frac{\mu_{nf}}{\mu_{f}ReDa}W-\frac{\rho_{nf}}{\rho_{f}}\frac{C_{F}}{\sqrt{Da}}\sqrt{U^{2}+V^{2}+W^{2}}\;W+\frac{{\left(\rho \beta \right)}_{nf}}{{\left(\rho \beta \right)}_{f}}\frac{Gr}{Re^{2}}\theta -\frac{\sigma_{nf}}{\sigma_{f}}Ha^{2}\;\frac{W}{\varepsilon }$$

(5) $$U\frac{\partial \theta }{\partial X}+V\frac{\partial \theta }{\partial Y}+W\frac{\partial \theta }{\partial Z}=\frac{{\left(\rho c_{p}\right)}_{f}}{{\left(\rho c_{p}\right)}_{nf}}\frac{k_{nf}}{k_{f}}\frac{1}{RePr}\left[\frac{\partial^{2}\theta }{\partial X^{2}}+\frac{\partial^{2}\theta }{\partial Y^{2}}+\frac{\partial^{2}\theta }{\partial Z^{2}}\right]$$

The following expressions denote the non-dimensional parameters:

(6) $$X,Y=\frac{x,y}{L},$$

(7) $$U,V,W=\frac{\left(u,v,w\right)L}{\alpha_{nf}},$$

(8) $$\theta =\frac{T-T_{c}}{T_{h}-T_{c}},\;$$

(9) $$Ra=\frac{g\beta_{fluid}\left(T_{h}-T_{c}\right)L^{3}}{\alpha_{fluid}v_{fluid}}$$

(10) $$P=\frac{\left(p\right)}{\rho_{nf}\frac{\alpha_{fl}^{2}}{L^{2}}}$$

(11) $$Da=\frac{K}{L^{2}}$$

(12) $$Ha=LB_{}\sqrt{\frac{\sigma_{nf}}{\mu_{nf}}}$$

(13) $$\mathrm{Pr}=\frac{v_{fluid}}{\alpha_{fluid}}$$

The governing equations’ boundary conditions read:

For Hypotenuse side walls:

(14) U=V=W=0, θ=0

For front and back walls:

(15) $$U=V=W=0,\;\frac{\partial \theta }{\partial n}=0$$

For the bottom wall:

(16) U=V=W=0, θ=1

Cylinder:

(17) $$U=-\Omega \left(Y-Y_{0}\right),V=\Omega \left(X-X_{0}\right),\frac{\partial \theta }{\partial Y}=0\;$$

The following correlations (Table 2) are utilized to determine the hybrid nanoliquid thermophysical properties:

Viscosity:

(28) $$\mu_{hnf}=\frac{\mu_{f}}{{\left(1-\varphi \right)}^{2.5}}$$

(29) $$\varphi =\varphi_{MWCNT}+\varphi_{Fe_{3}O_{4}}$$

The amount of entropy in a nanoliquid’s motion is predicted to change. Due to the fact that heat transfer is subject to specific variations, the total entropy may be characterized as follows [46]:

(30) $$S_{tot}=S_{ht}+S_{ff}+S_{mf}$$

(31) $$S_{ht}=\frac{k_{hnf}}{T_{0}^{2}}\left[{\left(\frac{\partial T}{\partial x}\right)}^{2}+{\left(\frac{\partial T}{\partial y}\right)}^{2}+{\left(\frac{\partial T}{\partial z}\right)}^{2}\right]$$

(32) $$\begin{array}{l} S_{ff}=\frac{\mu_{hnf}}{T_{0}}[2\left({\left(\frac{\partial u}{\partial x}\right)}^{2}+{\left(\frac{\partial v}{\partial y}\right)}^{2}+{\left(\frac{\partial w}{\partial z}\right)}^{2}\right)+{\left(\frac{\partial u}{\partial y}+\frac{\partial v}{\partial x}\right)}^{2}+{\left(\frac{\partial w}{\partial y}+\frac{\partial v}{\partial z}\right)}^{2}+{\left(\frac{\partial u}{\partial z}+\frac{\partial w}{\partial x}\right)}^{2}]\\ +\frac{\mu_{hnf}}{T_{0}K}\left(u^{2}+v^{2}+w^{2}\right) \end{array}$$

(33) $$S_{mf}=\frac{\sigma_{hnf}}{T_{0}}{\left(v\times B_{0}\right)}^{2}$$

$$\mathrm{with}\;T_{0}=\frac{T_{C}+T_{h}}{2}$$

Entropy generation *S_tot_* in non-dimensional form reads:

(34) $$S_{TOT}=S_{HT}+S_{FF}+S_{MF}$$

(35) $$S_{HT}=\frac{k_{hnf}}{k_{fluid}}\left[{\left(\frac{\partial \theta }{\partial X}\right)}^{2}+{\left(\frac{\partial \theta }{\partial Y}\right)}^{2}+{\left(\frac{\partial \theta }{\partial Z}\right)}^{2}\right]$$

(36) $$\begin{array}{l} S_{FF}=\frac{\mu_{hnf}}{\mu_{fluid}}N_{\mu }\left\{\begin{array}{l} \left[2{\left(\frac{\partial U}{\partial X}\right)}^{2}+2{\left(\frac{\partial V}{\partial Y}\right)}^{2}+2{\left(\frac{\partial W}{\partial Z}\right)}^{2}\right]\\ +{\left(\frac{\partial^{2}U}{\partial Y^{2}}+\frac{\partial^{2}V}{\partial X^{2}}\right)}^{2}+{\left(\frac{\partial^{2}W}{\partial Y^{2}}+\frac{\partial^{2}V}{\partial Z^{2}}\right)}^{2}+{\left(\frac{\partial^{2}U}{\partial Z^{2}}+\frac{\partial^{2}W}{\partial X^{2}}\right)}^{2} \end{array}\right\}\\ +\frac{\mu_{hnf}}{\mu_{fluid}}N_{\mu }\left(\frac{U^{2}+V^{2}+W^{2}}{Da}\right) \end{array}$$

(37) $$S_{MF}=N_{\mu }\frac{\sigma_{hnf}}{\sigma_{fluid}}Ha^{2}\left(U^{2}+V^{2}\right)$$

The $Nu_{loc}$ and $Nu_{avg}$ are estimated as:

(38) $$Nu_{loc}=-\frac{k_{eff}}{k_{fl}}{\left(\frac{\partial \theta_{po}}{\partial n}\right)}_{wall},Nu_{avg}=\frac{1}{S^{2}}\int_{0}^{s}\int_{0}^{s}Nu_{loc}dydz$$

## 3. Validation and Mesh Evaluation

**Figure 2:**
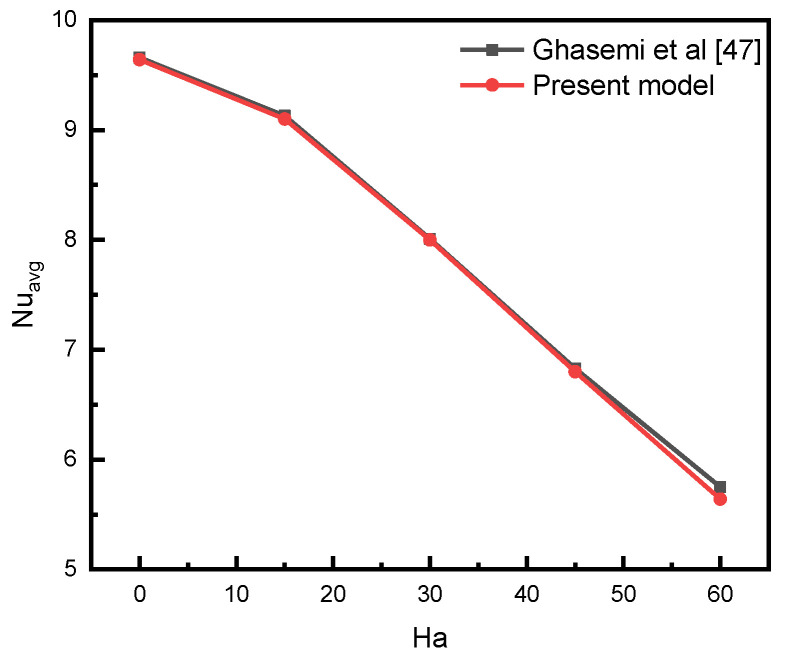
Comparisons of the present model with previous works [47]. Reproduced with permission from [47]. Elsevier, 2022.

The average Nusselt number (N_uavg_) and average Bejan numbers are used to demonstrate that heat transport is not dependent on the number of grids (see Table 3). Five different grids were utilized to ensure that the selected grid size did not influence the results. Due to the observed variability presented in Table 2, the fourth grid was selected as the final grid in all cases to keep reasonable computational cost while obtaining good precision of the result. As a primary criterion for arriving at conclusions, the numerical method used in the current study should be validated against the published literature. Previous investigations conducted by Ghasemi et al. [47] were utilized to validate the numerical model employed in the current study. As seen in Figure 2, our current model results agree well with the results presented by Ghasemi et al. [47].

It is crucial to keep in mind that the governing equations and their related constraints were solved by employing the Galerkin finite element technique. The programming environment is divided into triangle-shaped sections. Triangular Lagrange finite elements of various orders are employed on all the flow variables within the computational domain. By substituting the governing equations for the approximations, the residue is obtained.

$$\left|\frac{\Gamma^{i+1}-\Gamma^{i}}{\Gamma^{i}}\right|\leq \;10^{–6}$$

## 4. Results and discussion

The performance of the investigated cavity (see Figure 1) is discussed in this section, focusing on the flow and heat transfer behavior in a porous medium loaded with Fe_3_O_4_/MWCNT-water hybrid nanofluid. The cavity has an adiabatic cylinder that rotates in its core while the entire cavity is exposed to a magnetic field (from the cavity bottom). The hot bottom wall is undulated to ensure the further augmentation of heat transfer rates. The embedded cylinder is added to have a mixed heat transfer mechanism instead of the natural one. The results are represented in two schemes: massive visualizations (3D surface plots) to show the flow and heat transfer characteristics at specific conditions and 2D plots to show the parametric study of important variables. The selected variables are the Darcy number (to show the fluidity as a function of the porous media permeability), the angular velocity of the cylinder (to have mixed convection heat transfer), Hartmann number (to show the impact of the magnetic field, Lorentz force, over the flow viscosity), and the hot surface undulation number (to show any further enhancement in heat transfer rates). The effect of these variables is evaluated against the flow streamlines (to show the flow direction), dimensionless isotherms (to show the hot and cold regions), and Bejan number (as an assessment of the contribution of heat loss to the formation of total entropy). The investigated values of selected variables are −500 < Ω < 1000 rad/s for cylinder rotation speed, 0 < Ha < 100 for Hartmann number, *n* = 1, 2, 3, and 4 for the hot surface undulation number, and 0.01 < Da < 0.00001 for Darcy number.

### 4.1. Effect of a Cylinder Rotating Speed and Direction

**Figure 3:**
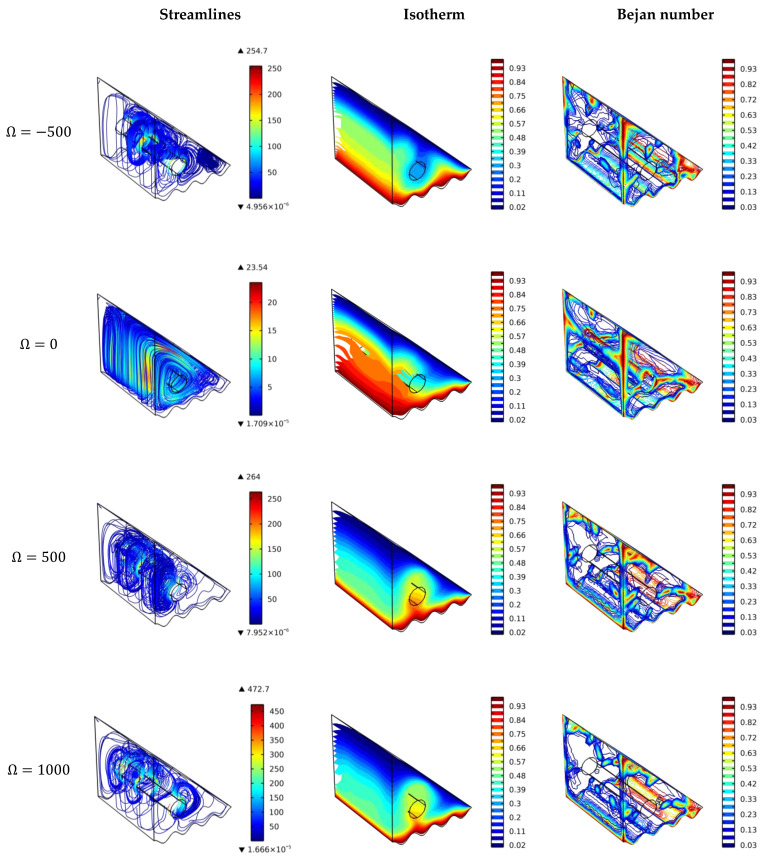
Surface plots of the velocity, temperature, and Bejan number for various cylinder speed values and directions.

Figure 3 shows the visualization plots of flow streamlines, isosurfaces of dimensionless temperature, and the local Bejan number for different cylinder rotating speeds, i.e., −500, 0, 500, and 1000. For Ω = 0 rad/s (no rotation); the streamlines show the smooth circulation of the nanofluids in the counter-clockwise direction, which is typically from the hot surface to the cold surface affected by the natural convection heat transfer. The hot nanofluid has a lower density so that it rises while the cold one takes its place, which is known as the buoyancy effect. The isotherms show a thick hot region at the left corner since the cold surface is far. For this case, the Bejan number values are higher at the surfaces and besides the cylinder as it is stationary. That is because heat transfer rates in these regions are low due to low turbulency (low fluid velocity).

The scenario is different for Ω = −500 and 500 rad/s, in which the mixed convection heat transfer is dominant and flow velocity increases, resulting in better mixing of hot and cold regions. For Ω = −500 rad/s (clockwise rotation), the heat transfer is better because the cold stream on the right side is mixed with the hot one and goes under the cylinder in the cavity bottom, and then it is enforced to rise up in the left side. After this, the mixed stream is circulated from left to right above the cylinder. This case (Ω = −500 rad/s) enhances the heat transfer rates except for the upper corner and some regions of the right corner where vortices (low motion) appear. These regions are far away from the rotating cylinder. The Bejan number is higher in these regions (due to low velocity), which means the heat losses contribute significantly (more than the flow friction) to the total entropy generation. For Ω = 500 rad/s (counter-clockwise rotation), the heat transfer is better above the cylinder (as it forces the mixing of hot and cold fluids to be above the cylinder) but worse under it compared to the case of clockwise rotation. It is obvious from these two cases (having the same cylinder rotation speed but different directions) that the direction of cylinder motion significantly affects the heat transfer rates. For Ω = 1000 rad/s, the heat transfer improves further by increasing the flow velocity and enhancing the hot and cold streams mixing. Furthermore, for Ω = –1000 rad/s (not shown in the figure), it is expected to have much better heat transfer (such as the case of Ω = −500 rad/s with a higher rotation speed). It should be noted that the blue regions in the Bejan number plots demonstrate the dominance of the flow friction on the entropy generation values where the heat losses are low.

### 4.2. Effect of Hartmann Number

**Figure 4:**
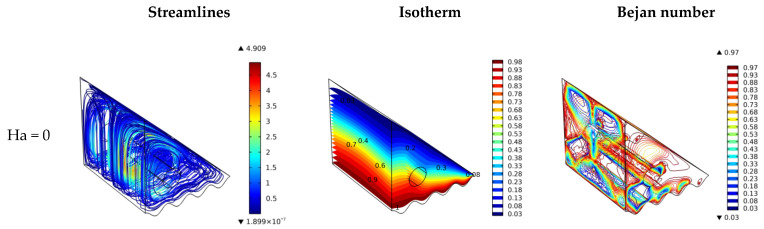

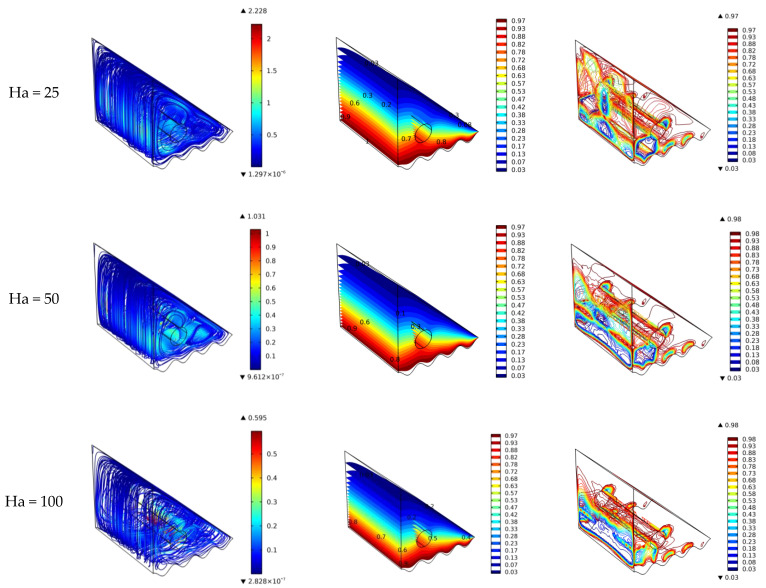
Surface plots of velocity, temperature, and Bejan number for various Hartmann numbers.

As metallic nanoparticles are used, the employment of magnetic fields from the bottom of the cavity to have upward hydromagnetic body force (Lorentz force) is expected to enhance heat transfer from the hot surface (at the bottom) to the cold surface (top and right side) because both act at the same direction. Still, simultaneously it hinders the recirculation of the cold stream from top to bottom (its effect and fluid flow are in the opposite direction). These two opposite impacts on the fluid movement could minimize the effect of the applied magnetic field. Furthermore, the cylinder rotation participates in flow movement and reduces the importance of the Lorentz force. However, the flow circulation might be hindered with higher Ha values such as Ha = 100, which has conflicting flow movements. Figure 4 confirms, in general, the lesser effect of the applied magnetic field on the flow and heat transfer in terms of streamlines, temperature features, and Bejan number. One could suggest having Ha = 0 for better fluid flow and heat transfer characteristics because the magnetic field has an opposite impact on the flow motion, resulting in an increase in the entropy generation.

### 4.3. Effect of Wavy Wall Undulation Number

**Figure 5:**
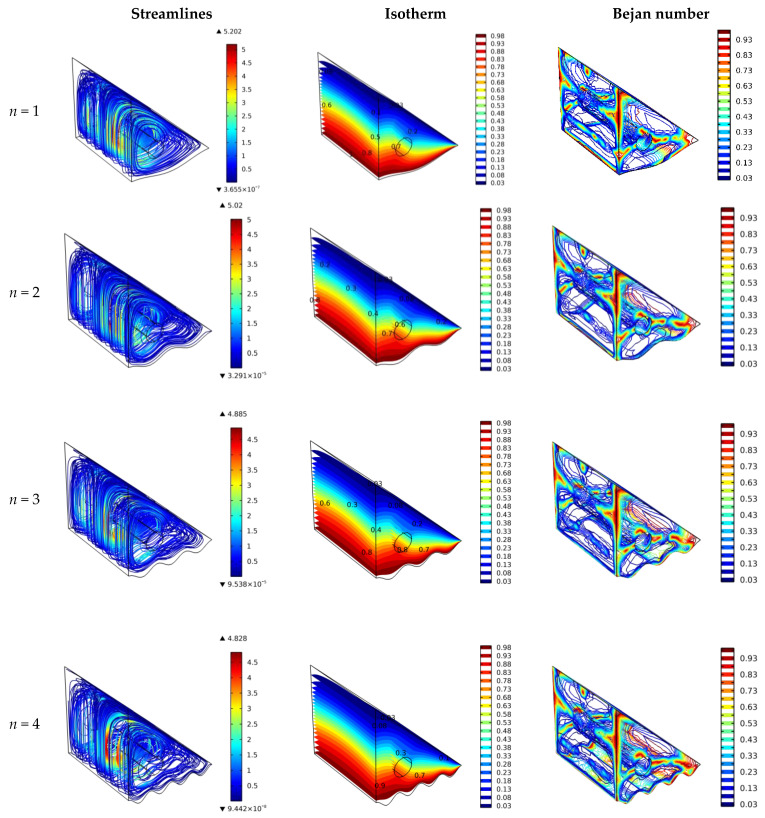
Surface plots of the velocity, temperature, and Bejan number for various wall undulation numbers.

The impact of the wavy wall undulation number on the results of streamlines, isotherms, and Bejan numbers is shown in Figure 5. It is shown that for *n* = 1, the streamlines are smoothly intensive with good heat transfer characteristics and a low Bejan number closer to the hot surfaces due to low heat transfer losses and better heat transfer rates. For the undulation number of ≥2, the flow friction is expected to increase closer to the hot surface, which might increase the heat transfer. The flow friction is shown by the lower streamline intensity for *n* of ≥2, while heat transfer does not improve much. This is because the mixed convection heat transfer could not exploit the roughness of the hot surfaces because the flow velocity closer to the wavy wall is not increased remarkably. Thus, more forces are required to have turbulent flow, such as those of forced convection heat transfer. The Bejan number values increase at the wavy surface for *n* ≥ 2 due to an increasing heat transfer area but without a significant improvement in heat transfer rates. Thus, the contribution of heat transfer in entropy generation is higher closer to the wavy surface of higher undulation.

### 4.4. Effect of Darcy Number

**Figure 6:**
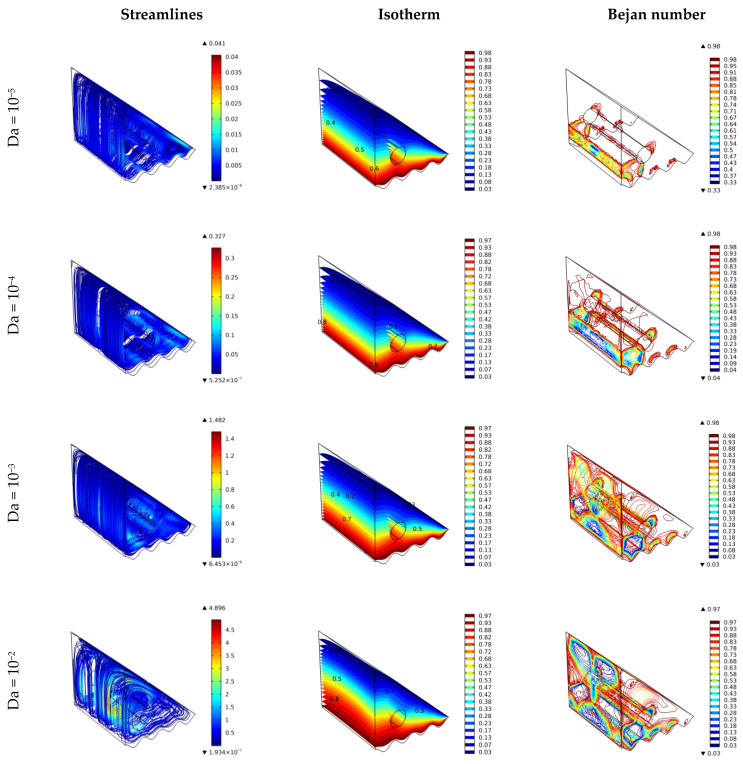
Surface plots of the velocity, temperature, and Bejan number for various Darcy number values.

As a function of the porous media permeability, the Darcy number dominates the flow of the investigated cavity. The influence of Da on stream function, temperature, and system entropy characteristics within a 3D cubic cavity is shown in Figure 6 for Da = 10^−5^, 10^−4^, 10^−3^, and 10^−2^. It is shown from the figure that streamlines and temperature distribution are better for higher values of Da (e.g., Da = 10^−2^). This is due to the increased permeability (low flow resistance from the porous media) and, therefore, more freedom for the nanofluids to flow (more fluidity). The low values of the Darcy number (Da < 10^−4^) increase the porous resistance and hence decrease the velocity and raise the pressure drop. The temperature characteristics imply that mixed convection is the primary mode of heat transfer for high Da numbers as flow velocity increases. At the same time, conduction is the predominant mode of heat transfer in the low Da. The Bejan number is reduced for increased Da due to enhancing the flow velocity and improved heat transfer.

### 4.5. Details Plots for the Parametric Study

**Figure 7:**
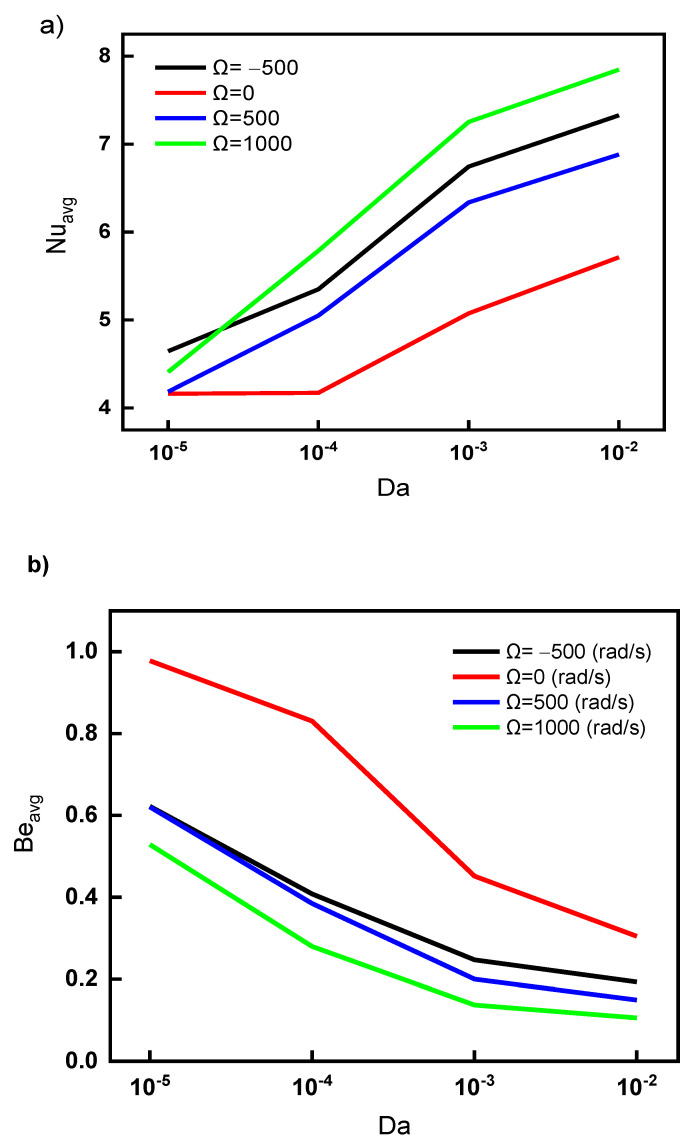
Impact of Darcy number on (**a**) Nu_avg_ and (**b**) Be_avg_ for various cylinder rotating speeds.

Figure 7 depicts the impact of Darcy number and cylinder rotating speed and direction on the heat transfer characteristics in terms of average Nusselt number (Figure 7a) and heat losses in terms of average Bejan number (Figure 7b). As evident, the increase in the Da number indicates a higher fluidity and low flow resistance. The flow circulation inside the cavity is due to the mixed convection; therefore, increasing flow blockage (reducing Da number) reduces the flow circulation and hence reduces Nu_avg_. Figure 7a clearly shows that Nu_avg_ is in direct proportion to the Da number; furthermore, increasing the rotational velocity of the cylinder enhances the flow circulation and improves *Nu_av_*_g_, especially for the clockwise direction (as Ω = −500 rad/s compared to Ω = 500 rad/s (counter-clockwise)), as described in some detail in Figure 3. The clockwise direction enforces cold streams mixing with the hot streams under the cylinder closer to the hot surface, and then the mixture (hot and cold streams) recirculates above the cylinder.

Figure 7b depicts the Bejan number behavior with different values of Da. It is clear that increasing the permeability of porous media enhances the heat transfer due to the increase in velocity, which minimizes heat losses, leading to a reduction in the Bejan number. Despite the better heat transfer, the case of Ω = −500 rad/s shows higher Bejan number values than of Ω = 500 rad/s due to increasing the heat losses at the corners of the cold surface (up and right sides) as the influence of the cylinder enforce the flow to go under the cylinder towards the insulated wall at the left of the cavity (for Ω = −500 rad/s). For Ω = 1000 rad/s and Da = 10^−2^, the enhancement in the average Nusselt number is about 38% and the drop in the Bejan number is 65% compared to the case of Ω = 0 rad/s and Da = 10^−5^.

**Figure 8:**
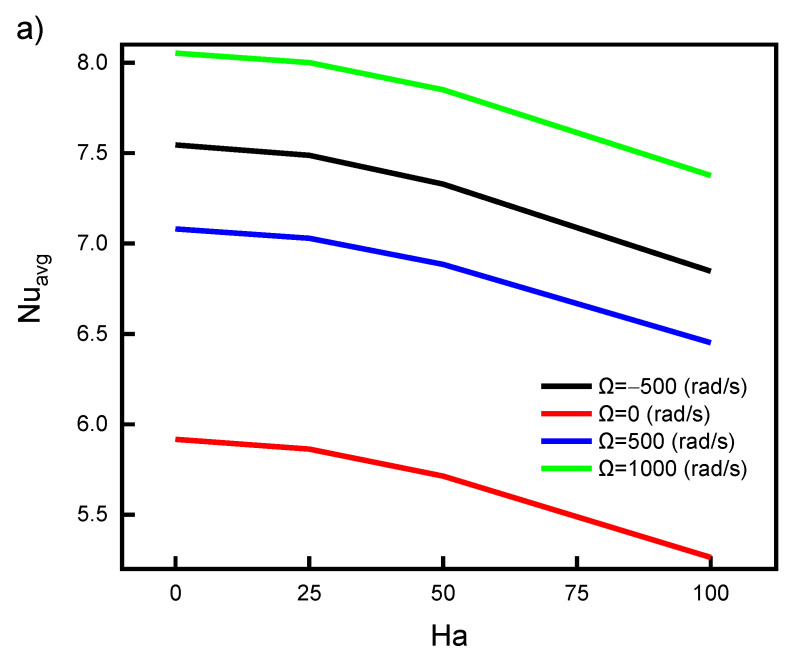

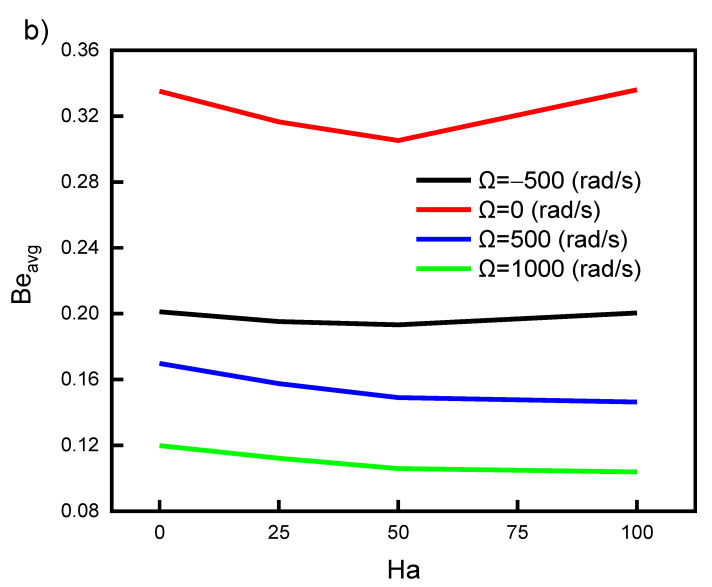
Impact of Hartmann number on (**a**) Nu_avg_ and (**b**)Be _avg_ for various cylinder rotating speeds.

The impact of the Ha number (Lorentz force) on Nu_avg_ and Be_avg_ is shown in Figure 8. The Lorentz force is applied in the same direction as the magnetic force is aligned with the positive z-direction. Along with the separation of the flow at the lower wavy hot wall, raising the Ha number resulted in the separation of the flow near the inclined cold wall. Therefore, increasing the Ha number decreases Nu_avg_, as shown in Figure 8a. This is because of the hindering of the flow recirculation process. It is also clear that Nu_avg_ is magnified by increasing the rotational velocity of the cylinder due to enhanced mixed convection heat transfer. As shown in Figure 8b, the impact of the Ha number on Be_avg_ is negligible, which does not mean the entropy generation is less, but the contributions of both friction and heat transfer are the same. At the same rotational speed, the Ha = 0 results in better Nu_aveg_ by 8% than at Ha = 100. One may recommend Ha = 0 and Ω = 1000 (or −1000) rad/s for higher heat transfer rates and lower entropy generation.

**Figure 9:**
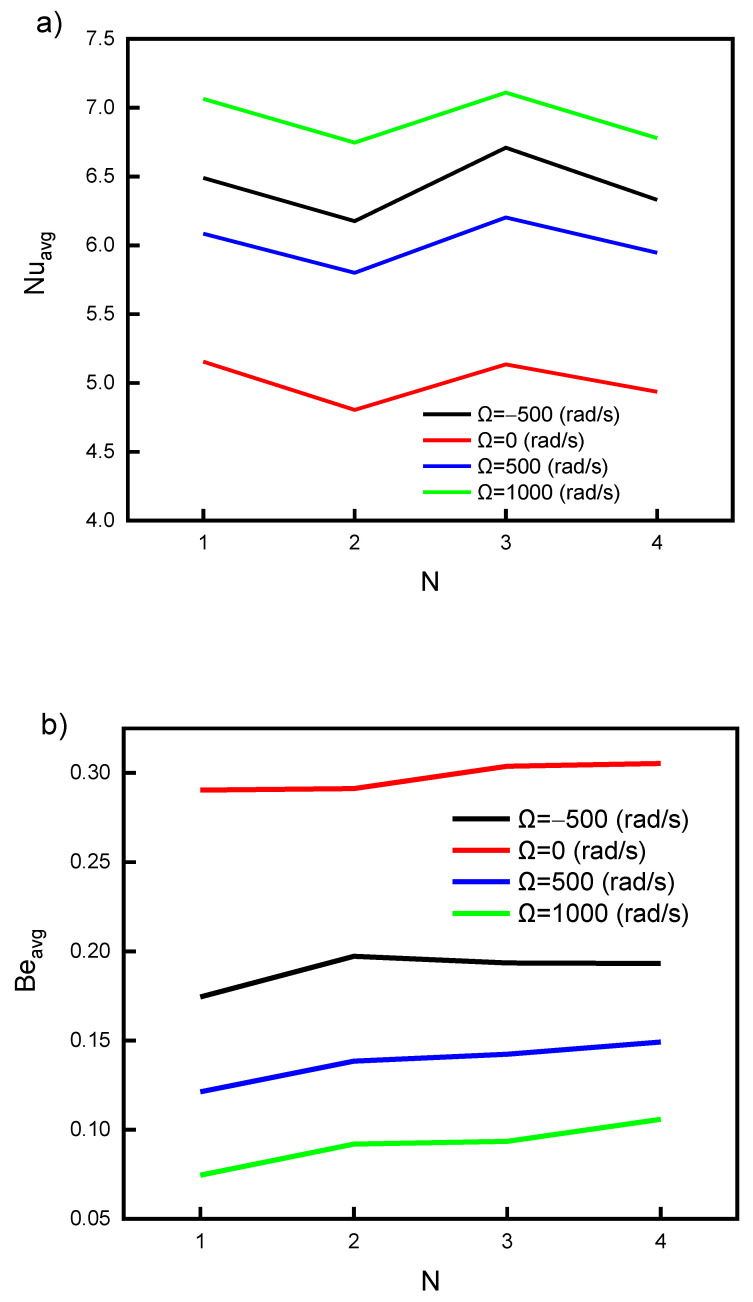
Influence of the number of undulations on (**a**) Nu_avg_ and (**b**) Be_avg_ for different cylinder rotating speeds.

Figure 9 illustrates the impact of the undulation number of the hot, wavy surface on the Nu_avg_ and Be_avg_. As discussed in Figure 5, the change in *n* value could not enhance the heat transfer rates (Nu values) when mixed convection is implemented. Thus, *n* = 1 is sufficient for such a case. The increment in *n* values could lead to significant turbulent flow for the forced heat transfer mechanism. The zigzag effect and little enhancement of Nu_avg_ values for *n* = 1 and 3 are due to the small vertical distance between the cylinder and wavy wall for these cases (as can be seen in Figure 5). The smaller distance between the cylinder and the wall results in a lower flow area and higher velocity. The investigated cases *n* = 2 and 4 have a wider distance between the cylinder and the projected point on the wavy surface. A wider distance means a higher flow area and lower turbulence, and therefore lower Nu values. Figure 9 shows no substantial change in the Nu_avg_ and Be_avg_ values. Hence, *n* = 1 is recommended for the investigated cavity. The biggest improvement (~40%) in Nu was obtained by rotating the cylinder at high speed (1000 rad/s).

It could be concluded from the discussed results that nanofluid flow and heat transfer characteristics in a porous medium could be enhanced for high Da, low Ha, *n* = 1, and high Ω values.

## 5. Conclusions

A three-dimensional triangular cavity filled with porous media and Fe_3_O_4_/MWCNT-water hybrid nanofluid is simulated in the current study. The bottom wall is hot and wavy, and the inclined lift wall is cold, while the other is insulated. The cavity is exposed to a magnetic field from the bottom in the positive z-direction. The flow region involves a revolving cylinder for the mixed convection heat transfer mechanism. The finite element method (FEM) is employed to solve the governing equations for fluid flow and heat transfer as a Newtonian flow. To analyze the fluid flow and heat transfer characteristics, some important parameters are investigated, such as Darcy number (10^−5^, 10^−4^, 10^−3^, 10^−2^), Hartmann number (Ha = 0, 20, 50, 100), cylinder revolution speed (Ω = −500, 0, 500, 1000 rad/s), and wavy wall undulation number (*n* = 1, 2, 3, and 4). The influence of these parameters is presented by 3D surface plots of streamlines, isotherms, Bejan number, and 2D profiles for the average Nusselt number and average Bejan number.

Based on the applied conditions, this study’s findings lead to the following conclusions:

Increasing the cylinder angular velocity enhances the nanofluid flow and heat transfer characteristics. A bigger enhancement is observed when the cylinder rotation direction is clockwise to enforce the cold streams mixed with the hot streams below the cylinder.

Increasing Ha values (magnetic effects) up to 100 hinders the flow motion and results in increased irreversibility due to both friction and heat transfer losses. However, due to the presence of the moving cylinder, the effect of the magnetic field is minimized.

Due to the resistance of the porous media, the fluidity and heat transfer are improved for higher Da values > 10^−3^.

The wavy wall undulation of *n* = 1 is sufficient for enhanced heat transfer and low irreversibility. More than 1 (*n* ≥ 2) has no discernible gain in heat transfer rates for mixed convection heat transfer.

Accordingly, the triangular porous cavity loaded with Fe_3_O_4_/MWCNT-water hybrid nanofluid could have enhanced heat transfer rates when Ha = 0, Da > 10^−3^, *n* = 1, and Ω > ± 500 rad/s.

## Figures and Tables

**Table 1:** Nanoparticles and base fluid thermos-physical properties [38,39].

	Pure Water	Fe_3_O_4_	MWCNT
ρ (kg/m^3^)	997.1	5180	2100
Cp (J/kg k)	4179	670	710
k (W/m k)	0.613	9.7	2000

**Table 2:** The correlations used to estimate the properties of classical and hybrid nanofluids [44,45].

Properties	Nanofluid Hybrid Nanofluid			
Density	$\rho_{hnf}=\left(1-\varphi \right)\rho_{fluid}+\varphi \rho_{hnp}$	(18)	$\rho_{hnp}=\frac{\varphi_{Fe_{3}O_{4}}\rho_{Fe_{3}O_{4}}+\varphi_{MWCNT}\rho_{MWCNT}}{\varphi }$	(19)
Heat capacity	${\left(\rho c_{p}\right)}_{hnf}=\left(1-\varphi \right){\left(\rho c_{p}\right)}_{fluid}+\varphi {\left(\rho c_{p}\right)}_{hnp}$	(20)	${\left(c_{p}\right)}_{hnp}=\frac{\varphi_{Fe_{3}O_{4}}{\left(c_{p}\right)}_{Fe_{3}O_{4}}+\varphi_{MWCNT}{\left(c_{p}\right)}_{MWCNT}}{\varphi }$	(21)
Thermal expansion coefficient	${\left(\rho \beta \right)}_{hnf}=\left(1-\varphi \right){\left(\rho \beta \right)}_{fluid}+\varphi {\left(\rho \beta \right)}_{hnp}$	(22)	$\beta_{hnp}=\frac{\varphi_{Fe_{3}O_{4}}\beta_{Fe_{3}O_{4}}+\varphi_{MWCNT}\beta_{MWCNT}}{\varphi }$	(23)
Electrical conductivity	$\sigma_{hnf}=\left(1-\varphi \right)\sigma_{fluid}+\varphi \sigma_{hnp}$	(24)	$\sigma_{hnp}=\frac{\varphi_{Fe_{3}O_{4}}\sigma_{Fe_{3}O_{4}}+\varphi_{MWCNT}\sigma_{MWCNT}}{\varphi }$	(25)
Thermal conductivity	$k_{hnf}=\frac{k_{hnp}+\left(n-1\right)k_{f}-\left(n-1\right)\left(k_{f}-k_{hnp}\right)\varphi }{k_{hnp}+\left(n-1\right)k_{f}+\left(k_{f}-k_{hnp}\right)\varphi }k_{f}$	(26)	$k_{hnp}=\frac{\varphi_{Fe_{3}O_{4}}k_{Fe_{3}O_{4}}+\varphi_{MWCNT}k_{MWCNT}}{\varphi }$	(27)

**Table 3:** Different mesh sizes for Ha = 0, Ω =0, and φ = 0.02.

**No. of Grid Elements**	**13,562**	**21,955**	**41,251**	**124,306**	**259,231**
Nu_avg_	6.7285	6.8965	7.3427	7.352	7.351
Be_avg_	0.29988	0.29566	0.29545	0.29544	0.29545

## Data Availability

The numerical data used to support the findings of this study are included within the article.
